# The management of lumbar intervertebral disc disease through siddha medicine - A case study

**DOI:** 10.6026/973206300223208

**Published:** 2026-05-31

**Authors:** Vishnu Priya K, Sugasini P, Jayachitra K, Sridevi J, Ganapathi R, Mahadevan M.V, Muthu Kumar N.J

**Affiliations:** 1 Department of Varma Maruthuvam, National Institute of Siddha, Chennai-47 (Affliated to The Tamilnadu Dr. M.G.R. Medical University, Chennai), Tamil Nadu, India; 2 Department of Siddhar Yoga Maruthuvam, National Institute of Siddha, Chennai-47 (Affliated to The Tamilnadu Dr. M.G.R. Medical University, Chennai), Tamil Nadu, India; 3 Department of Nanju Maruthuvam, National Institute of Siddha, Chennai-47 (Affliated to The Tamilnadu Dr. M.G.R. Medical University, Chennai), Tamil Nadu, India; 4 Department of Pura Maruthuvam, National Institute of Siddha, Chennai-47 (Affiliated to The Tamilnadu Dr. M.G.R. Medical University, Chennai), Tamil Nadu, India; 5 Director General, Central Institute for Research in Siddha, Chennai-47, Tamil Nadu, India

**Keywords:** Lumbar intervertebral disc disease, varmam therapy, external therapy

## Abstract

A herniated disc compresses the nerves or spinal cord causing pain and spinal cord dysfunction. This condition can be correlated with *Korai Vatham* mentioned in the Siddha text, *"Vaatha nithanam",* representing symptoms like Pain in the lumbo-sacral joint, heat and pricking sensation in the perineum, unable to sit erect, numbness or paraesthesia in the lower limbs. The patient, 34 years married male presented with complaints of pain in the low back region radiating to both lower limbs, and weakness in both lower limbs for the past 25 days was admitted in the IPD of the Varma maruthuvam Department in Ayothidoss Pandithar Hospital and treated with Varmam therapy and external therapies like *Ennai kattu, Kombaraku Otradam* along with internal medicine for 21 days. After the treatment of 21 days, the pain scoring in Oswestry Lowback Disability Scale was reduced from 37/50 to 22/50. This case study contributes to knowledge by aligning Siddha medicine with lumbar disc disease and presenting preliminary evidence that Varmam therapy, combined with external and internal Siddha treatments, can alleviate disability and enhance quality of life, as demonstrated through standardized outcome measures.

## Background:

Siddha medicine, one of India's oldest traditional systems, emphasises the balance of 3 Humours *(Vatham, Pitham and Kapham)* for maintaining health. *Varmam* therapy, a hallmark of Siddha, targets specific energy points to regulate life force *(Vaasi),* offer relief in musculoskeletal and neurological disorders [1]. One such condition is *Korai vaatham* (Lumbar Intervertebral disc disease), a degenerative disorder affecting the spinal discs. *Korai Vaatham,* corresponding to lumbar intervertebral disc disease (IVDD), is characterised by disc degeneration and nerve compression, leading to pain and neurological deficits. *Korai Vatham* mentioned in the *Siddha Varmam* text, *'Vaatha nithanam'*, representing symptoms like pain in the lumbo-sacral joint, heat and pricking sensation in the perineum, unable to sit erect, numbness or paraesthesia in the lower limbs. Treatment options vary depending on severity. Conservative approaches include rest, physiotherapy, pain management, and Siddha-based interventions like Varmam therapy to reduce inflammation and improve mobility [2, 3]. In severe cases, surgical procedures such as discectomy or spinal fusion may be necessary. If disc herniation becomes extreme, it can lead to Cauda Equina Syndrome (CES)-a rare but serious condition involving compression of the cauda equina nerves at the base of the spinal cord [4, 5]. CES presents with symptoms like lower limb weakness, saddle anesthesia, and loss of bladder or bowel control. It is a medical emergency requiring immediate surgical decompression to prevent permanent neurological damage. Early diagnosis and intervention are critical for recovery [6, 7 and 8]. Pooling of medicated oil in lumbar region *(Ennai Kattu)* and Heat fomentation *(Ottradam)* are traditional external therapies in Siddha medicine, used to treat musculoskeletal conditions. Therefore, it is of interest to report the management of Lumbar Intervertebral disc disease in Siddha system of medicine as a case report.

## Study type and intervention:

A case study design was adopted. A 34-year-old male patient who visited *Varma maruthuvam* OPD in Ayothidoss Pandithar hospital, National Institute of Siddha with the clinical features of Lumbar intervertebral disease in between the month of July 2022 was treated with *Varmam* therapy and other external therapies along with the internal medicines in the IP ward. The Lumbar intervertebral disease was diagnosed in these patients with the positive findings of Femoral Nerve Stretch test [8], diminished knee jerk and weakness in the both lower limb. The above patient was treated with the specific *Varmam* protocol along with *Ennai kattu, Kombuaraku otradam* and the IPD medicines. Each therapy was given for 7 consecutive days one followed by another.

## Clinical findings:

A 34-year-old married male who was working as a motor mechanic presented with complaints of pain in the low back region radiating to both lower limbs, and weakness in both lower limbs, unable to walk and use both lower limbs for the past 25 days. The patient had a history of heavy weight lifting followed by commencement of the symptoms. The patient was unable to walk and came to the OPD in a wheelchair. The patient was under Allopathic medication and physiotherapy treatment for the past 25 days. The patient was unsatisfied with the past treatment and reported to the Ayothidoss Pandithar Hospital. The patient had no history of bowel bladder disturbance, colour changes in lower limbs or trauma. The patient was admitted in the IPD of *Varmam* Department of Ayothidoss Pandithar Hospital, National Institute of Siddha after doing a clinical examination in the OPD. Sleep of the patient was disturbed due to the pain. The patient was non-vegetarian by diet and belonged to middle economic status with a normal physical built. The patient had no other co-morbid condition and family history related to this condition. The MRI taken after the day of impact revealed diffuse disc bulge in L1 - L2 intervertebral disc, broad postero-central protrusion causing ventral thecal sac indentation and mild narrowing of bilateral neural foramens, diffuse disc bulge in L2 - L3 intervertebral disc, broad postero-central and bilateral paracentral nucleus pulpous protrusion and extrusion causing cauda equina nerve roots indentation and encroachment of bilateral intervertebral foramens.

## Diagnostic assessments:

The pain and disability were assessed by Oswestry Low Back Disability Scale [9]. From analysis of the above symptoms and clinical findings, he was diagnosed to be affected by Lumbar Intervertebral disc disease.

## Examination in siddha accepts:

The body constitution *(Yaakkai ilakkanam*) of the patient was Kaba vatha thegi. On examining humours *(Uyir thathukal)*, in *Vaatham,* Viyanan, *Samanan* were affected, in *Pitham, Saathaga pitham* was affected and in *Kabam, Santhigam* was affected. In *Udal thathukal, Saaram, Kozhuppu, Enbu* were affected.

## Therapeutic interventions:

## Treatment protocol:

The patient underwent a 21-day regimen including: Ennai Kattu with *Laguvidamutti Thylam* for 7 days, Varmam Therapy targeting 11 specific points, *Kombarakku Ottradam* (herbal fomentation) for 7 days, Internal Medicines: Amukkara *Chooranam, Ayaveera Chenduram and Kukkil Parpam* and Dietary Restrictions: Anti-Vatha diet

## Warm medicated oil pooling (Ennai kattu):

In this procedure the medicated oil is poured over the affected area (Lumbar region) in an enclosed built *(Puravalayam)* made of Urad dal flour. The *Laguvidamusti thylam* (medicated oil) is heated and poured in warm state over the affected area. *Ennai kattu* was done with the *Laguvidamutti thylam* for about 45 minutes for 7 consecutive days.

## Siddha Varmam therapy:

The *Varmam* therapy was given regularly for 7 consecutive days. The *Varmam* therapy was applied in lying and sitting position. Pressure was given with the fingers of the physician with the specified unit *(Maathirai*) as mentioned below table. The duration of each *Varmam* therapy session was 15 minutes. The details of the *Varmam* points were mentioned in the Table 2.

## Kombaraku otradam:

The *Kombarakku otradam* [13] was given regularly for 7 consecutive days. The *Kombarakku otradam* was done in lying position. The *Kombarakku otradam* was made as an Herbal pouches (Kizhi) and heated in the ghee. After heating in the ghee, the Kizhi is placed over the affected area. The ingredients were powdered in equal quantities and made into bundles (Kizhi). This therapeutic bundle is heated in the cow ghee and used for fomentation. The fomentation is given over the mudichi region, along the spine, over the back muscles, low back region and lower limbs. The *Kombarakku otradam* is indicated for stiffness, pricking pain, muscle spasms and sprain in the Varmam literature, *"Varma odivu murivu sara soothiram* - 1102/1200". The ingredients of the *Kombarakku otradam* were mentioned in the Table 2.

## Concomitant medication:

## Internal medication:

Pathiyam (Diet):

The Anti-Vatha diet was advised for the patients during the treatment period. The anti- Vatha diet represents restriction of tuber varieties, vegetables like Pumpkin *(Poosani)*, Calabash *(Surai)*, Ribbed gourd *(Peerku)*, Snake gourd *(Pudalai)*, Legumes, Horse gram, Corn, Tamarind and spicy foods. The patient is instructed to avoid Tobacco chewing and Alcohol.

## Outcome measure:

After the treatment of 21 days, the intensity of the pain was reduced. The pain scoring in Oswestry Low back Disability Scale was reduced from 37/50 to 22/50. Clinical examination revealed improved range of motion in the lumbar spine and diminished paraspinal muscle tenderness. Neurological assessment remained stable, with no signs of motor or sensory deficits.

## Data analysis:

The demographic data of the patient was recorded. The difference in the outcome was reported by total scores and percentage of improvement before and after the study.

## Results:

After undergoing a 21-day course of treatment, the patient experienced a marked reduction in pain intensity and functional impairment associated with low back pain. This improvement is reflected in the Oswestry Low Back Disability Scale score, which decreased significantly from 37 out of 50 (indicating a 74% disability level) to 22 out of 50 (44% disability level). Such a reduction suggests a transition from severe to moderate disability, highlighting enhanced mobility, reduced discomfort during daily activities, and an overall positive response to the therapeutic intervention. The outcome underscores the effectiveness of the treatment in improving the patient's quality of life and functional capacity.

## Follow up and outcomes:

At the one-month follow-up, the patient reported notable improvements in symptoms associated with intervertebral disc disease. Pain intensity had decreased significantly, with fewer episodes of radiating discomfort and reduced reliance on analgesics. Functional mobility improved, allowing the patient to perform daily activities with greater ease and less restriction. The patient adhered well to the prescribed core strengthening exercises, postural correction, Simple *yogasanam, Pranayamam,* and ergonomic training [18].

## Discussion:

This case study highlights the integrative potential of Siddha medicine in managing lumbar intervertebral disc disease (IVDD), traditionally known as *Korai Vaatham.* The patient presented with classical signs of disc herniation and nerve root compression, including radiating pain, motor weakness, and sensory deficit -symptoms that align closely with descriptions in *Vaatha Nithanam.* The therapeutic sequence was strategically designed as mentioned in Table 6. *Ennai Kattu*, administered initially, served to reduce local inflammation and muscle spasm. The ingredients of *Laguvidamutti Thylam* mentioned in the Table 3, with known anti-inflammatory and analgesic properties, likely facilitated soft tissue relaxation and improved local circulation [17]. Varmam therapy, the cornerstone of Siddha neuro-musculoskeletal management, was employed to stimulate specific energy points *(adangal)* along the spine and lower limbs. The selected points as mentioned in the Table 4 were chosen based on their anatomical relevance to nerve pathways and their traditional indications for pain modulation and energy flow correction. The gentle, rhythmic stimulation using calibrated pressure *(maathirai)* likely contributed to neuromuscular rebalancing and pain relief [18, 19 and 20]. Following *Varmam, Kombarakku Ottradam* was applied to reinforce therapeutic effects. The herbal fomentation, as mentioned in Table 5, containing ingredients like *Cateria lacca, Nardostachys jatamansi,* and *Zingiber officinale,* provided deep tissue heating, enhanced transdermal absorption, and further reduced inflammation [13]. The synergy between Varmam and Ottradam reflects the Siddha principle of sequential therapy, where one modality potentiates the next. The internal medications complemented the external therapies. *Amukkara Chooranam* is known for its adaptogenic and neuroprotective effects [14], Ayaveera Chenduram for its anti-inflammatory action, [15] and *Kungiliya Parpam* for its analgesic and detoxifying properties [16]. Together, they addressed the systemic Vatha derangement and supported tissue regeneration. The significant reduction in ODI score (from 74% to 44%) not only demonstrates symptomatic relief but also functional recovery. This is clinically meaningful and there is significant reduction in the clinical symptoms as mentioned in Table 2, as a 10-15 point drop in ODI is considered a threshold for substantial improvement. The treatment also made a noteworthy change in the Eight-fold Siddha examination as shown in the Table 1. The patient's ability to transition from wheelchair-bound immobility to independent ambulation within three weeks underscores the therapeutic value of the Siddha protocol. At one-month follow-up, the absence of neurological deterioration and sustained functional gains suggest that the intervention had both immediate and lasting benefits. The patient's adherence to post-discharge physiotherapy and dietary guidelines likely contributed to this outcome.

## Conclusion:

We show the relevance of Siddha medicine particularly Varmam therapy in managing complex neuromuscular disorders like *Korai vatham* (Lumbar Intervertebral Disc Disease). It also illustrates the importance of individualized, multimodal treatment strategies that address both symptomatic and root-level imbalances. While this is a single case, the results warrant further investigation through controlled clinical trials to validate efficacy and explore mechanisms of action.

## Patient perspectives:

The patient self-reported that he was satisfied with the treatment as he experienced reduction in pain and weakness in both lower limbs. His quality of life was improved.

## Informed consent:

The patients were well informed about the Varmam therapy, Ennai kattu and Otradam. The informed consent was obtained from the patient before starting the treatment. The patient has given consent for images and other clinical information to be reported in the journal. The patient was assured that his name and personal information will not be published and efforts will be made to conceal his identity.

## Source of funding:

None

## Figures and Tables

**Table 1:** Siddha therapeutic envakai thervu (eight-fold) assessment [10 ]

**Siddha assessment**	**Before treatment**	**After treatment**	**Follow-up**
Na (tongue examination)	Coated tongue	Moist, mild coated tongue	Moist, mild coated tongue
Niram (colour of the body)	Maaniram (Tan colour)	Maaniram (Tan colour)	Maaniram (Tan colour)
Moli (speech)	Low pitched	Normal pitched	Normal pitched
Vili (eye)	Normal	Normal	Normal
Sparicam (palpation)	Dry/Lustreless, mild warmth present in the low back region	Good texture skin with no warmth in low back region	Good texture skin with no warmth in low back region
Nati (Pulse)	Vāta pittam	Pitta vatam	Pitta vatam
Malam (motion examination)	Constipated	Freely passing stool	Freely passing stool
Muttiram (urine examination)	Yellowish urine	Pale yellowish urine	Pale yellowish urine
Neykkuri (Oil on urine sign)	Spread like snake and disperse as ring pattern	Ring with elongated edges pattern	Ring pattern

**Table 2:** Clinical examination [11]

**Neurological assessment**	**Before treatment**	**After treatment**	**Follow-up**
Inspection	No swelling or redness. No deformity or muscle wasting	No swelling or redness. No deformity or muscle wasting	No swelling or redness. No deformity or muscle wasting
Tenderness	Tenderness was present in the low back region, slight sensory deficit (Tingling numbness) felt in the lateral aspect of both legs	Tenderness present in the low back region reduced, slight sensory deficit (Tingling numbness) felt in the lateral aspect of both legs improved.	No Tenderness present in the low back region, No sensory deficit (Tingling numbness).
Movement	Hip joint: All the movements of the hip were affected.	Hip joint: All the movements of the hip were improved.	Hip joint: Can do all movements of the hip.
	Knee joint: Normal	Knee joint: Normal	Knee joint: Normal
	Ankle joint: Normal	Ankle joint: Normal	Ankle joint: Normal
Nutrition	Normal	Normal	Normal
Power	Grade 3 in both lower limbs	Grade 4 in both lower limbs	Grade 5
Tone	Slightly Hypotonic in lower limbs	Slightly Hypotonic in lower limbs reduced	Normal
Reflex	Diminished knee and Ankle reflex	Diminished knee and Ankle reflex improved	Normal
Sensory examination	Pain: Diminished in both lower limbs	Patient able to feel the pain, touch and temperature	Normal
	Touch: Diminished in both lower limbs		
	Temperature: Diminished in both lower limbs		

**Table 3:** Ingredients of laguvidamutti thylam

**S.No**	**Ingredients**	**Botanical name**
1	Ettikottai	Strychnos nux-vomica
2	Poondu	Allium sativum
3	Aayil thool	Holoptelea integrifolia
4	Nalla ennai	Sesamum indicum
5	Vellattupaal	Capra aegagrus

**Table 4:** Details of Varmam therapy [12, 13]

**S.NO**	**Adangal**	**Anatomical location**	**Patient position**	**Unit of pressure (maathirai)**	**Procedure**
1	Mudichi 5		Lying position	¼ maathirai	Clockwise
	1. Karunathi mudichi	Atlanto occipital joint			And anti-clockwise rotation is made in each mudichi and the energy transmitted to the succeeding point without taking hand. Repeat this for three times.
	2. Sara mudichi	C7-T1 joint space			
	3. Thunnal mudichi	T8 -T9 joint space			
	4. Pathi-pasu-paasa mudichi	Lumbo sacral joint			
	5. Kumbaga Mudichi	Tip of the coccyx			
2	Nanganaar pootu adangal	Present at the sacro iliac joint 2 finger width lateral to the gluteal cleft	Lying position	¼ maathirai	Simultaneous pressure in both the points and ended laterally
3	Putti poruthu adangal	In between the inferior part of gluteus and Posterior part of the thigh below the ischial tuberosity	Lying position	¼ maathirai	Simultaneous pressure in both the points and lifted in the upward direction
4	Amani Varmam	Centre of anterior part of thigh	Lying or sitting position	¼ maathirai	Simultaneous Clockwise rotation in both points and lifted in the upward direction
5	Kal mootu varmam	Present in the center of popliteal fossa	Lying or sitting position	¼ maathirai	Simultaneous pressure in the varmam point with middle finger of both hands.
6	Komberi kalam	Present 8 fingers above medial malleolus	Lying or sitting position	½ maathirai	The varmam point is stimulated with middle three fingers in the upward direction.
7	Kuthikal adangal	Insertion point of achilles tendon on calcaneal bone	Lying or sitting position	¼ maathirai	Pressure is given in the thumb and index finger upward and downward direction
8	Viruthi adangal	In the base of the great toe in the dorsal aspect	Lying or sitting position	½ maathirai	Pressure is given with the thumb finger in upward direction
9	Puliyamuthu adangal	Present at the base of great toe	Lying or sitting position	½ maathirai	Pressure is give with the thumb finger in downward direction
10	Ullangal vellai adangal	Centre part of the foot	Lying or sitting position	½ maathirai	Pressure is give with the thumb finger in upward direction
11	Uchi pathaippu	8 fingers width above the thilartha varmam	Sitting position	1 maathirai	Left palm is placed 3 finger above this point and tapped with the right hand over it.

**Table 5:** Ingredients of Kombarakku otradam [13]

**S.No**	**Ingredients**	**Botanical name**
1	Kombarakku	Cateria lacca
2	Sadamanjil	Nardostachys jatamansi
3	Vaal milagu	Piper cubeba
4	Chukku	Zingiber officinale
5	Pasu nei	Cow ghee

**Table 6:** Therapeutic intervention

**S. No**	**Siddha drug**	**Dosage/Interval**	**Anubanam (Adjuvant)**	**Days**
1	Agastiyar kulambu	200 mg/OD	Ginger juice	One day
2	Amukkara chooranam	2 gm/BD	Warm water	21 days
3	Kukkil parpam	200 mg/BD	Milk	21 days
4	Ayaveera chenduram	100 mg/BD	Honey	21 days
6	Kunthiriga thailam	50 ml/SOS	-	21 days
